# The association between duration of nicotine pouch use and oral mucosal lesions in male dental students and interns: A cross-sectional study

**DOI:** 10.18332/tid/224437

**Published:** 2026-08-05

**Authors:** Sarah AlFarabi Ali, Deema Sahab, Ayman Bukhari, Malek Baobied, Soulafa AlMazrooa, Sara Akeel

**Affiliations:** 1 Oral Diagnostic Sciences Department, Faculty of Dentistry, King Abdulaziz University, Jeddah, Saudi Arabia; 2 Department Public Health, Faculty of Dentistry, King Abdulaziz University, Jeddah, Saudi Arabia; 3 BDS, Faculty of Dentistry, King Abdulaziz University, Jeddah, Saudi Arabia

**Keywords:** nicotine pouch, noncombustible products, nicotine-containing products, oral mucosal lesion, white lesion

## Abstract

**INTRODUCTION:**

The increasing popularity of oral nicotine pouches (ONPs) has raised significant public health concerns regarding their impact on oral health. These products deliver nicotine through mucosal membranes without combustion, prompting the need for comprehensive research into their effects on the oral mucosa. The objective of this study was to investigate the association between duration of nicotine pouch use and oral mucosal lesion severity.

**METHODS:**

This was a cross-sectional study of a convenience sample of 42 male students and interns at King Abdulaziz University Faculty of Dentistry (KAUFD), Saudi Arabia, who used ONPs. Clinical examinations were conducted to assess the severity of oral mucosal lesions, which were categorized ordinally. Duration of ONP use was measured using a combined index [duration (months) × frequency (pouches/day)]. Ordinal logistic regression was used to evaluate the association between duration of ONP use and severity of oral mucosal lesions, adjusting for age, smoking status, and pouch placement behavior.

**RESULTS:**

In adjusted analyses, oral mucosal lesion severity was not significantly associated with duration of ONP use (AOR=1.001; 95% CI: 1.000–1.002, p=0.161), age (AOR=0.77; 95% CI 0.21–2.87, p=0.697), or smoking status (AOR=0.68; 95% CI: 0.20–2.25, p=0.523).

**CONCLUSIONS:**

Oral mucosal lesion severity was not associated with the duration or frequency of ONP use. Future research should focus on examining the long-term effects of nicotine pouch use on oral health to better understand and mitigate health risks.

## INTRODUCTION

Oral nicotine pouches (ONPs) are small, tobacco-free bags filled with nicotine and other additives. Unlike traditional smokeless tobacco (SLT) products (such as dip or chewing tobacco), ONPs do not include tobacco leaf. While some manufacturers advertise ONPs as a safer replacement for smoking or dipping, they are not FDA-approved as a nicotine replacement therapy^1^. ONPs typically contain nicotine, water, flavorings, sweeteners, and plant-based fibers^2^. They are small, pre-measured sachets that resemble traditional snus in both appearance and method of use, as they are placed between the gum and lip^2^. However, unlike conventional SLT, which is made from processed tobacco leaf, ONPs contain either synthetic or tobacco-derived nicotine incorporated into a white powdered base or a plant fiber substrate, so they are frequently marketed as ‘tobacco-free’^2^. An ONP is placed between the gum and inner lip for 30–60 minutes and is not chewed, smoked, or swallowed. Instead, nicotine is absorbed through the oral mucous membranes into the bloodstream^1^.

There is evidence that ONP use is increasing in various demographic groups, not least adolescents, raising concerns about potential nicotine addiction and related health concerns in younger people. For example, 3.4% of high school students in Southern California reported using flavored non-tobacco oral nicotine products, including ONPs^3^. In another study, 35% to 42% of a sample of Polish adolescents were aware of ONPs, with many considering them less harmful than combustible cigarettes. This perception, together with their appealing flavors, could contribute to increasing use in younger people^4^.

The popularity of ONPs is driven by a combination of flavor appeal, convenience, and the perception that they pose less harm than combustible tobacco. Survey evidence suggests that users develop substantial nicotine dependence and frequently report mouth lesions as the most common adverse event, with mint and tobacco flavors emerging as the principal drivers of use; these patterns have prompted calls for regulatory oversight of flavor formulations from a public health perspective^5^.

Prolonged nicotine exposure, irrespective of delivery route, has been associated with a range of oral health problems^6^. Reported mucosal effects of ONPs include homogeneous white lesions at the placement site with histological features of parakeratosis and chronic inflammatory infiltrate^7^, and an increased frequency of gum bleeding and ulcerative lesions in adolescent users^8^. Conversely, replacing tobacco-based pouches with non-tobacco ONPs has been shown to reduce pre-existing oral mucosal lesions over short follow-up^9^, indicating that local mucosal effects, while common, vary substantially with product composition.

Cumulative exposure also appears to influence oral health outcomes. Daily and high-frequency users of snus and ONPs report a higher burden of oral health problems than occasional users, raising concerns about the effects of extended use^10^. Although ONPs may carry a lower overall risk than combustible tobacco^11^, local mucosal and periodontal effects, including inflammation and dysbiosis, remain a clinically relevant concern^12^. Together, this emerging evidence has led several authors to recommend limiting the duration and frequency of ONP use until safer thresholds can be established.

Therefore, the growing popularity of ONPs has prompted a public health debate. Given their increasing use across diverse populations, including in young people, it is crucial to examine their safety and potential negative effects on the oral mucosa^2^. Current evidence in Saudi Arabia primarily consists of surveys assessing prevalence, usage patterns, and awareness and perceptions of ONPs in adults^13-17^. ONP use appears to be higher in males, younger adults, and in individuals who use other tobacco products^14,16,17^. However, none of these studies examined the relationship between ONPs and oral mucosal lesions. Additionally, although several studies reported self-perceived health symptoms^14,16^, none used clinical investigations to assess oral or general health outcomes related to ONP use. Accordingly, we hypothesized that a longer duration of ONP use is significantly associated with a greater severity of oral mucosa lesions.

The aim of this cross-sectional study was to investigate the association between duration of ONP use (specifically DZRT, a local tobacco-free pouch) and the severity of oral mucosal lesions in a convenience-based sample of male students and interns attending King Abdulaziz University Faculty of Dentistry (KAUFD), Jeddah, Saudi Arabia.

## METHODS

### Study population and design

This was a cross-sectional study performed in compliance with relevant laws and institutional guidelines. The Research Ethics Committee, Faculty of Dentistry, King Abdulaziz University approved the study protocol (REC no.128-11-24). Participants were recruited as a convenience sample from King Abdulaziz University Faculty of Dentistry (KAUFD) between January and March 2025. Eligible participants were male students or interns who were using ONP at the time of data collection. Exclusion criteria included students who used other smokeless tobacco products, topical products, or medications known to affect the oral mucosa.

**Figure 1:**
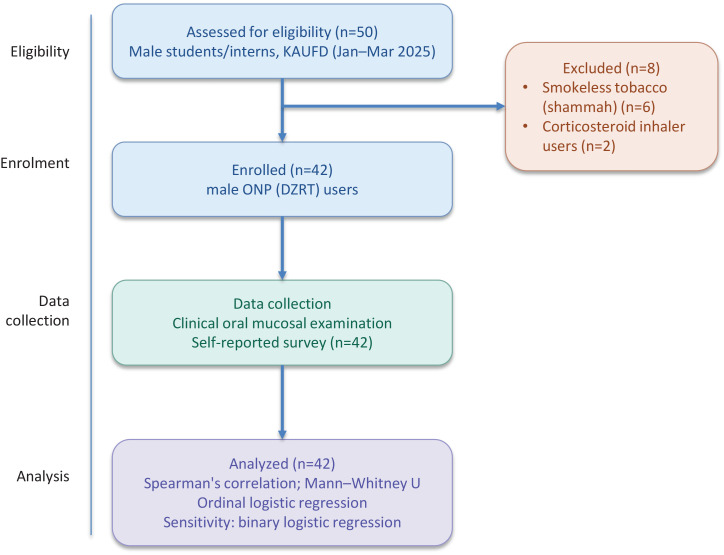
Participant recruitment, screening, and exclusions in a cross-sectional study of male dental students and interns who used oral nicotine pouches at King Abdulaziz University Faculty of Dentistry (KAUFD), Jeddah, Saudi Arabia, January–March 2025 (N=42)

Participants provided informed consent, and data were collected through clinical examinations and a previously published self-reported survey^18^. Clinical assessment involved visual inspection of the oral mucosa, while the survey included questions on demographics, nicotine usage patterns, and self-reported oral hygiene habits. Fifty male students between second and internship years at KAUFD who used ONPs were considered eligible for inclusion (specifically DZRT) (Figure 1).

### Sample size calculation

Sample size was calculated using G*Power based on detecting an association between duration of oral nicotine pouch use and oral mucosal lesions, with 80% power and a 5% significance level. Due to the limited availability of prior quantitative estimates on the association between duration of oral nicotine pouch use and oral mucosal lesions, the effect size for the sample size calculation was based on an assumed moderate association (OR=1.5). Based on these assumptions, the estimated sample size was 170 participants.

### Assessment metrics

**Figure 2:**
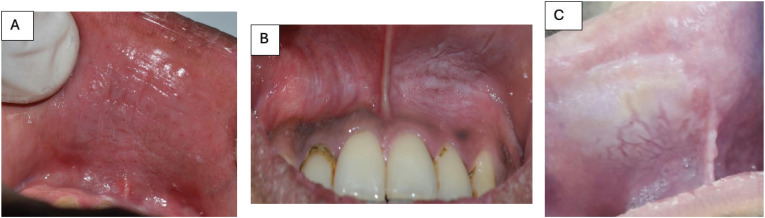
Assessment of smokeless tobacco keratosis (STK) severity in male dental students and interns who used oral nicotine pouches at King Abdulaziz University Faculty of Dentistry (KAUFD), Jeddah, Saudi Arabia, January–March 2025 (N=42): A) Degree I STK, superficial keratosis with slight opaqueness score 1; B) Degree II STK, white keratosis with moderate wrinkling score; C) Degree III STK, white lesions with intervening furrows

Clinical examiners assisted participants in completing the survey and conducted clinical examinations to assess oral mucosal lesions using standardized assessment tools by Greer and Poulson^19^. A modified Axell grading system for smokeless tobacco keratosis (STK) was used^20^, which categorizes STK lesions into three severity categories: Degree I lesion, characterized as superficially keratotic, exhibiting slight opaqueness, a color similar to the surrounding mucosa, slight wrinkling, and no obvious mucosal thickening (Figure 2A); Degree II lesion, also superficial keratosis, typically appearing white with occasional reddish areas, moderate wrinkling, and lacking obvious or dramatic thickening (Figure 2B); and Degree III lesion, present as a white lesion with intervening furrows of normal mucosal color, clear mucosal thickening, and dramatic wrinkling (Figure 2C).

### Outcomes

The primary outcome was oral mucosal lesion severity, classified into ordered categories based on clinical appearance and severity as: 0=no lesions, 1=Degree I lesion, 2=Degree II lesion, and 3=Degree III lesion.

### Exposures

The exposure variable was duration of ONP use, which was a cumulative-exposure proxy by multiplying history of use (months) by daily frequency (pouches per day), in keeping with the pack-years and smokeless-tobacco cumulative-exposure metrics widely used in tobacco research^21^.

### Potential confounders

Covariates were selected a priori based on biological plausibility and existing literature and included age, smoking status, and consistency of pouch placement site.

Age was categorized according to the standard age-group classifications used by the General Authority for Statistics (GASTAT) in Saudi Arabia (aged 18–24 years and 25–29 years) to maintain consistency with nationally recognized demographic reporting and facilitate comparability with national datasets^22^.

Smoking status was recorded as never (individuals who reported never using tobacco), former (those who had previously used tobacco but had quit at the time of the study), occasional individuals who reported using tobacco irregularly or not on a daily basis), and daily smoker (those who reported consistent, everyday use). However, due to sparse data in some categories, smoking status was recoded into a binary variable representing current smoking (occasional or daily smoker) versus not currently smoking (never or former smoker).

Consistency of pouch placement site was assessed by whether the participant consistently placed the nicotine pouch in the same intra-oral site (yes, no).

### Statistical analysis

Data were analyzed using Stata/SE 15.1 (StataCorp, College Station, TX, USA). Descriptive statistics were used to summarize participant characteristics and study variables. Categorical variables (oral mucosal lesion severity, age groups, smoking status, and oral nicotine pouch placement behavior) are presented as frequencies and percentages. Continuous variables (history of oral nicotine pouch in months and frequency of use as number of pouches per day) are summarized using medians and interquartile ranges (IQRs).

Correlations between oral mucosal lesion severity and continuous or categorical exposure variables (history, frequency, duration index, age) were examined using Spearman’s rank correlation coefficient. Correlation strength was interpreted as weak (|ρ| <0.30), moderate (0.30–0.59), strong (0.60–0.79), or very strong (≥0.80). Associations between oral mucosal lesion severity and binary exposure variables (current smoking status and consistency pouch placement site) were assessed using Mann–Whitney U tests.

Ordinal logistic regression was used to model the association between oral mucosal lesion severity and ONP use. The proportional odds assumption for ordinal logistic regression was assessed using a generalized ordered logistic regression model. This assumption was satisfied for all predictors. Outcome category frequencies were examined to assess model stability. Multicollinearity was evaluated using pairwise correlation coefficients. History and frequency of ONP use were initially examined separately; however, due to collinearity between them (r=0.765), they were not entered simultaneously into the multivariable model. Instead, a proxy exposure duration index (history × frequency) was used in the final model. The final multivariable model included cumulative ONP duration, age category, consistency of pouch placement site, and current smoking status. As a sensitivity analysis, the outcome was dichotomized into the presence or absence of oral lesions (yes, no) and analyzed using binary logistic regression to assess the robustness of the observed associations and overcome the paucity of observations in some of the categories in the ordinal model. Results are presented as odds ratios (ORs) with 95% confidence intervals (CIs). A p<0.05 was considered significant.

## RESULTS

### Participants characteristics

A total of 50 participants were recruited, and only 42 participants were included in the analysis (Figure 1). Six participants were excluded because they used other smokeless tobacco products (shammah), and two more participants were excluded because they used corticosteroid inhalers, which are known to affect the oral mucosa^23,24^. Students were aged 18–29 years, with 71% of the sample (30 students) aged <24 years and 29% (12 students) aged 25–29 years. Nineteen participants (45.2%) had no oral mucosal lesions, sixteen participants (38.1%) had Degree I lesions, five (11.9%) had Degree II lesions, and 2 (4.8%) participants had Degree III lesions.

Duration of ONP use ranged between 2 and 96 months, with a median 11 months (IQR: 6–12). The number of ONPs used per day (frequency of use) ranged between 1 and 30 pouches, with a median 5.5 pouches per day (IQR: 3–7). Regarding the history of tobacco smoking, 4.8% (n=2) were never smokers, 45% (n=19) were previous smokers, 14% (n=6) smoked occasionally, and 35.7% (n=15) smoked daily. Most participants (81%, n=34) placed the pouches in different places, while 19% (n=8) placed the pouch consistently in the same place intra-orally. Participant details are shown in Table 1.

**Table 1:** Characteristics of male dental students and interns who used oral nicotine pouches at King Abdulaziz University Faculty of Dentistry (KAUFD), Jeddah, Saudi Arabia, January–March 2025 (N=42)

*Characteristics*	*n*	*%*
Oral mucosal lesion severity*		
No lesion	19	45.2
Degree I lesion	16	38.1
Degree II lesion	5	11.9
Degree III lesion	2	4.8
**History of ONP use** (months), median (IQR) range	11 (6–12) 2–96	
**Frequency of ONP use** (pouches/day), median (IQR) range	5.5 (3–7) 1–30	
**Age** (years)		
18–24	30	71.4
25–29	12	28.6
**Smoking**		
Not currently smoking	21	50
Currently smoking	21	50
**Consistency of ONP placement site**		
Different places	34	80.95
Same place	8	19.05

* Degree I lesion: superficially keratotic, exhibiting slight opaqueness, a color similar to the surrounding mucosa, slight wrinkling, and no obvious mucosal thickening (Figure 2A). Degree II lesion: superficial keratosis, typically appearing white with occasional reddish areas, moderate wrinkling, and lacking obvious or dramatic thickening (Figure 2B). Degree III lesion: white lesion with intervening furrows of normal mucosal color, clear mucosal thickening, and dramatic wrinkling (Figure 2C). IQR: interquartile range.

### Predictors of oral mucosal lesion severity

Spearman’s rank correlation analysis showed a weak non-significant correlation between the outcome (oral mucosal lesion severity) and history of ONP use (ρ= -0.048, p=0.764), frequency of oral nicotine pouch use (ρ=0.233, p=0.137), age (ρ= -0.090, p=0.573), and duration index (ρ=0.110, p=0.578) (Table 2).

**Table 2:** Univariate analyses of predictors of oral mucosal lesion severity in a cross-sectional study of male dental students and interns who used oral nicotine pouches at King Abdulaziz University Faculty of Dentistry (KAUFD), Jeddah, Saudi Arabia, January–March 2025 (N=42)

*Variables*	*Test*	*Statistic*	*p*
**History of ONP use** (months)	Spearman’s rank correlation	ρ= -0.048	0.764
**Frequency of ONP use** (pouches/day)	Spearman’s rank correlation	ρ=0.233	0.137
**Duration of ONP use** (history × frequency)	Spearman’s rank correlation	ρ=0.110	0.578
**Age**	Spearman’s rank correlation	ρ= -0.090	0.573
**Current smoking status**	Mann–Whitney U tests	z=0.293	0.775
**Consistency of ONP placement site**	Mann–Whitney U tests	z=1.980	0.048

Mann–Whitney U tests showed no significant difference in oral mucosal lesion severity between current and non-current smokers (z=0.29, p=0.775). There was a marginally statistically significant relationship between oral mucosal lesion severity and consistency of nicotine pouch placement (z=1.98, p=0.048), with higher lesion severity observed among participants who did not consistently place the pouch in the same location (Table 2).

Univariate ordinal logistic regression showed no statistically significant association between mucosal lesion severity and history of nicotine pouch use, frequency of use, ONP duration, age, or smoking status. A negative association was observed between lesion severity and consistent placement of nicotine pouches at the same intra-oral site, where participants who consistently placed the pouch in the same location had lower odds of higher lesion severity (OR=0.19; 95% CI: 0.03–1.05), although this association did not reach statistical significance (p=0.057) (Table 3).

**Table 3:** Ordinal logistic regression analysis of predictors of oral mucosal lesion severity in a cross-sectional study of male dental students and interns who used oral nicotine pouches at King Abdulaziz University Faculty of Dentistry (KAUFD), Jeddah, Saudi Arabia, January–March 2025 (N=42)

*Variables*	*OR (95% CI)*	*p*	*AOR (95% CI)*	*p*
**Duration of ONP use** (history × frequency)	1.001 (1.000–1.002)	0.133	1.001 (1.000–1.002)	0.161
**Age** (years)				
18–24 (ref.)	1.000		1.000	
25–29	0.688 (0.194–2.446)	0.564	0.770 (0.206–2.87)	0.697
**Current smoking status**				
Not currently smoking (ref.)	1.000		1.000	
Currently smoking	0.843 (0.269–2.646)	0.771	0.675 (0.202–2.254)	0.523
**Consistency of ONP placement site**				
Different places (ref.)	1.000		1.000	
Same place	0.189 (0.034–1.052)	0.057	0.194 (0.0341–1.103)	0.064

AOR: adjusted odds ratio; derived from a multivariable ordinal logistic regression model that included duration of ONP use (history × frequency), age category, current smoking status, and consistency of ONP placement site as predictors. Statistical significance was set at p<0.05.

Multivariable ordinal logistic regression including duration of ONP exposure, age categories, consistency of pouch placement, and current smoking status showed that none of the predictors was significantly associated with lesion severity within our population. Severity of oral mucosal lesions was not significantly associated with duration of ONP use (AOR=1.001; 95% CI: 1.000–1.002, p=0.161), consistent pouch placement in the same intra-oral site (AOR=0.194; 95% CI: 0.034–1.103, p=0.064), smoking status (AOR=0.675, 95% CI: 0.202–2.254, p=0.523), or age category (AOR=0.770; 95% CI: 0.206–2.872, p=0.697) (Table 3). Multivariable binary logistic regression analysis, conducted as a sensitivity analysis and with the presence of oral mucosal lesions (yes, no) as an endpoint, showed similar non-significant results (Supplementary file Table 1).

## DISCUSSION

This study found that oral mucosal changes were relatively common among ONP users, with over half of participants showing some degree of lesion severity, although most lesions were mild. Despite variability in duration, frequency, and cumulative exposure to ONPs, no significant association was identified between these measures and mucosal lesion severity within our population. Age and smoking status were also not significantly associated with lesion severity in this sample. Interestingly, consistent placement of ONPs at the same intra-oral site showed an inverse association with lesion severity in univariable analysis and a similar non-significant trend in the multivariable model, suggesting that placement behavior may warrant further investigation. Overall, the findings indicate that, within this relatively small and predominantly short-term user sample, ONP exposure was not clearly associated with greater oral mucosal lesion severity, highlighting the need for larger studies with longer follow-up and more detailed assessment of product use patterns. These results address a knowledge gap on the health effects of nicotine pouches on oral health; specifically, the association between the severity of the mucosal change and the duration of nicotine pouch use and pouch placement behavior in Saudi Arabia.

The increasing popularity of ONPs represents a significant change in nicotine consumption, especially among younger people, who often see them as safer alternatives to traditional tobacco products^25^. Smoking and smokeless tobacco are known to have detrimental effects on periodontal health through mechanical irritation and friction from the pouch, while chemical irritation from ONP constituents may alter epithelial cell turnover, thereby promoting para/ hyperkeratosis^26,27^. Furthermore, mucosal healing may be impaired, and low-grade inflammation may occur due to nicotine-mediated vasoconstriction and immune modulation^7,28^.

The existing literature indicates that prolonged nicotine exposure, regardless of the administration approach, can cause oral health problems^29^. Indeed, chronic use of smokeless tobacco products has been linked to oral mucosal lesions, inflammation, and other negative health effects, but study designs, product types, and exposure metrics vary and sometimes produce differing effect estimates. For instance, mucosal changes were reported in users who used ONPs and snus, especially those with a longer duration of use and frequent consumption of 5–10 pouches per day^7^.

Similarly, over half the participants in this study had oral mucosa lesions. However, there was no statistically significant association between mucosal lesion severity and duration of nicotine pouch use, daily frequency of use, or total nicotine pouch exposure. Our data are consistent with a controlled clinical study of snus-like non-tobacco nicotine pouches, which reported that non-tobacco pouches did not exacerbate pre-existing lesions and, in some settings, were associated with decreased lesion severity over six weeks compared with tobacco-containing snus, suggesting product composition strongly influences outcomes^9^. A case series with histopathological analysis documented white, keratotic, and parakeratotic lesions at pouch sites with ONP use, supporting a causal biological effect at the local tissue level^7^. Mechanistically, the oral mucosa is permeable, so irritants can more readily penetrate at these highly vascularized sites^30^. Furthermore, the chemical mixtures in ONP and the mucosa can interact by being irritative or allergenic, promoting mucosal inflammation. Tissue reactivity may be influenced by the amount of exposure, the irritative potential of the chemical constituents, and the penetrative capability of toxins^5^. Contradicting our study, Rungraungrayabkul et al.^31^ reported that oral mucosa changes are common in ONP users, and they seem to be related to ONP units consumed per day and to their duration of usage; we detected no association between cumulative exposure and lesion severity, even though oral mucosal changes were common. The varying concentrations of tobacco and flavors in ONP may have an impact on mucosal responses^32^. However, as our sample size was relatively small and the duration of use was shorter than in previous reports, further studies are warranted to explore the relationship between exposure and mucosal severity.

Interestingly, another study found no differences between users of snus and ONPs. These results suggest that those using ONP may be at higher risk of developing white oral mucosal lesions in areas of product placement compared with other tobacco or nicotine delivery systems^6,31^. We detected a weak and borderline significant trend towards a negative influence of consistent pouch placement on lesion severity, contrary to previous studies. However, we did not include the duration of single use in the oral cavity for each placement, which may have contributed to the observed negative result. Over half of the participants practicing consistent placement of ONPs showed Degree I lesions, but other factors such as the concentration of the product, flavor, and host inflammatory response may have influenced the mucosal response. Our study suggests the possibility that oral mucosal lesion severity may not be associated with the duration or frequency of ONP use alone, but may be additionally influenced by placement behavior rather than cumulative exposure. As many studies report on the amount of ONP used and the duration of use rather than site and placement consistency, further research is needed to examine the impact of placement behavior on lesion development. Furthermore, there is a need to investigate the range of tobacco-free ONP products available in the market, as the effects are variable^33^.

Taken together, our analyses produced null findings: neither duration of ONP use, frequency of use, nor cumulative exposure was significantly associated with oral mucosal lesion severity, and the inverse signal observed for consistent pouch placement did not reach statistical significance after adjustment. Several factors are likely to have contributed to these null results. Statistical power was limited; with only 42 participants recruited against a calculated requirement of 170, the study was underpowered to detect associations of the magnitude assumed *a priori*, increasing the likelihood of type II error. Exposure was also predominantly short-term, with 80% of participants having used ONPs for less than one year, so any cumulative effect on the mucosa may not yet have manifested. Finally, several biologically relevant aspects of exposure were not captured at sufficient granularity, including the per session intra-oral contact time of each pouch, the nicotine concentration of the specific DZRT product used, and flavor formulation, all of which may modify mucosal response and could therefore have attenuated the observed associations

### Limitations

This study has some limitations. A main limitation was the sampling strategy, which was convenience-based and cross-sectional. Participants were selected based on accessibility rather than random selection from the target population. Consequently, the sample may not fully represent the broader population, and the observed associations should be interpreted with caution.

Due to the cross-sectional design, exposure and outcome were assessed at the same time point; therefore, temporality could not be established. Although the estimated required sample size was 170 participants to achieve 80% power, only 42 participants were recruited, reducing the statistical power and increasing the possibility of type II error. This relatively small sample was largely due to the limited number of ONP users within the university setting from which participants were recruited. It is also important to note that the study population was limited to male students enrolled at the Faculty of Dentistry. As such, the findings are intended to reflect this specific population rather than the broader population of ONP users. While this may limit generalizability, the findings of this study contribute early evidence on the potential association between duration of use and oral mucosal lesion severity in a real-world setting. Furthermore, participants’ prior knowledge of the potential effects of ONP on the oral mucosa may have influenced their pattern of use and consequently the outcomes of the study. Additionally, there was bias towards individuals with a shorter history of ONP use. Indeed, 80% of the sample had used nicotine pouches for less than one year, and it is possible that the effects of ONPs on oral health may develop over a longer period. The findings highlight the feasibility of recruiting and studying this population but also the need for larger, multi-center studies to further investigate these associations. In this context, the study serves as a foundation for future research by generating hypotheses, informing effect size estimates, and identifying key variables for inclusion in subsequent, adequately powered studies.

Because ONP use patterns were based on selfreported data, the findings may be subject to recall and reporting bias. Misclassification of exposure variables such as duration, frequency, placement behavior, and smoking history may have occurred, which may have attenuated the observed associations with oral mucosal lesion severity. Of note, lesion assessment relied on clinical visual inspection; it is important to maintain clinical calibration to ensure lesion grading when multiple assessors are involved. Residual confounding factors to consider include product heterogeneity, exposure metrics differences, and user behavior: oral hygiene practices and dietary habits all impact outcomes^9,10^. An important but unmeasured factor to consider includes the duration of ONP application (the time a pouch is kept intra-orally in every use), as this could be an important factor in measuring the duration of exposure. Of note, there are currently no comparable studies on DZRT and their association with oral mucosa lesions in the literature.

### Implications

Considering our findings and reports reflecting mucosal changes in response to ONP use, we recommend that dental professionals ask about ONP use, inspect pouch placement sites for keratotic changes, counsel users about potential local effects, and encourage limiting per-session contact until more definitive evidence becomes available.

### Future research

Future research should focus on examining the long-term effects of nicotine pouch use on oral health to better understand and mitigate health risks. This is especially important for vulnerable groups, such as adolescents, who may view these products as safer options than traditional tobacco. As nicotine pouches continue to gain traction, particularly among young people, it is crucial that health education campaigns accurately communicate the risks linked to their use.

## CONCLUSIONS

Our study contributes to the understanding of the health implications associated with ONP use. Our study suggests that oral mucosal lesion severity was not associated with the duration or frequency of ONP use within our population and may be influenced by placement behavior rather than cumulative exposure. Future cohort studies with larger sample sizes should focus on elucidating the long-term effects of nicotine pouch use, aiming to inform public health policies and promote safer usage practices to mitigate potential health risks.

## Supplementary Material



## Data Availability

The data supporting this research are available from the authors on reasonable request.
