# Supplementary material for: The association between duration of nicotine pouch use and oral mucosal lesions in male dental students and interns: A cross-sectional study
Source: Tob Induc Dis. 2026 Aug 5;24:10.18332/tid/224437. doi: 10.18332/tid/224437 (PMC13450131; doi:10.18332/tid/224437)
Supplement: Supplementary file 1 [file TID-24-141-s1.pdf]

***Supplementary Table 1. Multivariable binary regression analysis of predictors of lesion severity in a cross-sectional study of male dental students and interns who used oral nicotine pouches at King Abdulaziz University Faculty of Dentistry (KAUFD), Jeddah, Saudi Arabia, January–March 2025 (n=42)***

| | **Adjusted Odds Ratios [95% CI]** | ***p*-value** |
|---|---|---|
| **Total ONP exposure** (duration x frequency) | 1.001 [0.998 – 1.004] | 0.518 |
| **Age** | | |
| 18-24 years | 1.000 [reference] | - |
| 25-29 years | 0.813 [0.190 – 3.482] | 0.780 |
| **Current smoking status** | | |
| Not currently smoking | 1.000 [reference] | - |
| Currently smoking | 0.413 [0.107 – 1.593] | 0.199 |
| **Site of nicotine pouch placement** | | |
| Different places | 1.000 [reference] | - |
| Same place | 0.183 [0.029 - 1.132] | 0.068 |

Adjusted odds ratios are derived from a multivariable ordinal logistic regression model that included duration of ONP use (history × frequency), age category, current smoking status, and consistency of ONP placement site as predictors. Statistical significance was set at $p < 0.05$.

The content has been provided by the author(s) and has not been reviewed, verified, or endorsed by European Publishing. It may not have undergone peer review. The views, opinions, and recommendations expressed are solely those of the author(s) and do not necessarily reflect the position of European Publishing. European Publishing accepts no responsibility or liability for any consequences arising from the use of, or reliance on, this content.

© 2026 Ali S. A. et. al. DOI:10.18332/tid/224437
